# Mitral stenosis severity and the hypoxic response: potential role of hypoxia-inducible factor-2α - a cross-sectional study

**DOI:** 10.1186/s12872-026-05744-6

**Published:** 2026-03-16

**Authors:** Meltem Uyaner Kan, Hasan Kan, Yakup Alsancak, Filiz Alkan Baylan

**Affiliations:** 1 Department of Medical Biochemistry, Niğde Ömer Halisdemir University Training and Research Hospital, Niğde, Türkiye; 2 Department of Cardiology, Niğde Ömer Halisdemir University Training and Research Hospital, Niğde, Türkiye; 3 Department of Cardiology, Necmettin Erbakan University, Konya, Türkiye; 4 Department of Medical Biochemistry, Necmettin Erbakan University, Konya, Türkiye

**Keywords:** Mitral stenosis, EPAS1, HIF-2α, Pulmonary hypertension, Biomarker, Hypoxia-inducible pathways

## Abstract

**Background:**

Mitral stenosis (MS) causes chronic pressure overload and secondary pulmonary hypertension, which are associated with activation of hypoxia-inducible pathways. This study investigated the circulating levels of hypoxia-inducible factor-2α (Endothelial PAS domain protein 1-EPAS1) in patients with MS and their relationship with disease severity.

**Methods:**

This prospective study included 69 patients diagnosed with rheumatic MS and an equal number of age- and sex-matched healthy individuals. Hypoxia-inducible factor-2α (HIF-2α) levels were assessed using an enzyme-linked immunosorbent assay (ELISA) along with conventional biomarkers, and all patients underwent echocardiographic assessments. Statistical comparisons were performed using appropriate parametric or non-parametric tests, and relationships were evaluated using Spearman’s rank correlation coefficients.

**Results:**

HIF-2α levels were significantly higher in patients with MS than in the controls (median 5.2 vs. 4.7; *p* = 0.0275). Patients with MS also had higher high-sensitivity cardiac troponin T levels (median 7.7 vs. 4.9, *p* = 0.0328) than controls. HIF-2α was weakly positively correlated with systolic pulmonary artery pressure (ρ = 0.28, *p* = 0.001), left atrial diameter (ρ = 0.22, *p* = 0.012), and N-terminal pro-B-type natriuretic peptide level (ρ = 0.29, *p* = 0.014). HIF-2α levels did not significantly correlate with the mitral valve area or mean transmitral gradient.

**Conclusion:**

Elevated HIF-2α levels in patients with MS are correlated with pulmonary hypertension and cardiac stress. These findings support the involvement of hypoxia-inducible pathways in the pathophysiology of MS. However, HIF-2α elevation is modest and its clinical utility as a biomarker for MS is currently limited.

## Background

Mitral stenosis (MS) involves constriction of the mitral valve orifice, resulting in increased pressure and enlargement of the left atrium, eventually leading to pulmonary venous hypertension. Over time, it can progress to pulmonary arterial hypertension and right ventricular overload, thereby causing considerable cardiovascular and pulmonary complications. The severity of MS is clinically and echocardiographically assessed using parameters such as mitral valve area (MVA), transmitral pressure gradient, and pulmonary artery pressure (PAP). However, biochemical and molecular mechanisms, particularly those related to chronic hypoxia in the pulmonary circuit and myocardium, remain incompletely understood.

Hypoxia-inducible factor-2α (HIF-2α), also referred to as Endothelial PAS domain protein 1 (EPAS1), is a transcription factor that is crucial for regulating cellular reactions to hypoxia [1]. Under low-oxygen conditions, HIF-2α is stabilized and translocated to the nucleus to activate hypoxia-responsive genes. Hypoxia-inducible factors are central regulators of vascular homeostasis and remodelling, particularly under chronic hypoxic stress [2]. Endothelial HIF-2α has been implicated in maladaptive vascular responses [3].

There is evidence that hypoxia signalling is involved in valvular pathology. In vitro, mitral valve interstitial cells exposed to hypoxic conditions stabilize HIF-1α and upregulate matrix metalloproteinases, leading to extracellular matrix remodelling [4]. Similarly, HIF-2α and NF-κB are highly expressed alongside angiogenesis and collagen remodelling markers in stenotic aortic valves [5], suggesting that chronic valvular diseases such as MS may locally activate hypoxia-inducible factor (HIF) pathways and contribute to progressive fibrosis and valve deformation.

We propose that in rheumatic mitral stenosis, elevated pulmonary pressure induces endothelial hypoxic activation and the HIF-driven release of pro-fibrotic mediators. Given its role in hypoxic pulmonary hypertension [6], HIF-2α is a potential biomarker for MS. Therefore, this study aimed to quantify circulating HIF-2α levels in patients with mitral stenosis and to assess their correlation with disease severity.

## Methods

### Study design and participants

This prospective study, conducted between May and July 2025, included 69 consecutive patients aged > 18 years with echocardiographically confirmed rheumatic mitral stenosis referred for evaluation and 69 age- and sex-matched healthy controls with no evidence of cardiac pathology on echocardiography. Participants were excluded if they had any comorbid conditions known to influence HIF-2α levels, including active malignancy, chronic inflammatory or infectious diseases, significant hepatic dysfunction, chronic neurological or musculoskeletal disorders, chronic kidney or respiratory disease, thyroid dysfunction, adrenal disorders, or gonadal hormone disorders. Patients with more than mild coexisting valvular lesions or a reduced left ventricular ejection fraction were also excluded to isolate the effects of MS.

### Data collection and measurements

After enrolment, demographic data and clinical history were recorded. All participants underwent transthoracic echocardiography (Philips Medical Systems, Andover, MA, USA) performed by an experienced cardiologist. For patients with MS, key echocardiographic measurements included the mitral valve area (planimetry in the parasternal short-axis view), transmitral pressure gradients (via Doppler), systolic pulmonary artery pressure (PAP) estimated from the peak tricuspid regurgitant velocity plus right atrial pressure, and left atrial diameter. The severity of mitral stenosis and related echocardiographic parameters were assessed according to the American Society of Echocardiography Guidelines for the Evaluation of Rheumatic Heart Disease [7]. In the control group, echocardiography confirmed a normal cardiac chamber size, normal valves (mitral valve area typically > 4 cm²), and normal estimated PAP.

Venous blood samples were collected from each participant after overnight fasting. Samples were immediately centrifuged at 1500 × g for 15 min, and the separated serum was aliquoted into Eppendorf tubes and stored at − 80 °C until analysis. A human HIF-2α enzyme-linked immunosorbent assay kit (Bioassay Technology Laboratory, China, Cat. No: E7164Hu) was used for serum measurements. Each sample was analyzed in a single technical measurement according to the manufacturer’s protocol, with internal quality controls included in each run. To mitigate batch effects in the measurements, all samples were processed within a single session. Enzyme-linked immunosorbent assay (ELISA) plates were read at 450 nm using an Epoch microplate reader (BioTek Instruments, Inc., Winooski, VT, USA), and HIF-2α concentrations were calculated from the standard curve. The analytical sensitivity of this kit was 0.044 ng/ml, with intra- and inter-assay coefficients of variation < 8% and < 10%, respectively.

Serum hs-cTnT and NT-proBNP levels were measured using an electrochemiluminescence immunoassay (Cobas 8000 e801; Roche Diagnostics, Mannheim, Germany), HbA1c with Premier Hb9210 (Trinity Biotech, Bray, Ireland), and a complete blood count using Sysmex XN-1000 (Sysmex Corporation, Kobe, Japan). Fasting glucose, urea, creatinine, and albumin levels were assessed using a Cobas 8000 c702 analyzer (Roche Diagnostics, Mannheim, Germany).

### Statistical analysis

Statistical analyses were performed using IBM SPSS Statistics version 26 (IBM Corp., Armonk, NY, USA) and GraphPad Prism version 9.2 (GraphPad Software, San Diego, CA, USA). Normal distribution was assessed using the Kolmogorov-Smirnov test. Descriptive statistics for continuous variables are provided as either mean with standard deviation (SD) or median with interquartile range (IQR), based on their distribution. For categorical variables, these statistics are represented as counts and percentages.

To compare the MS and control groups, either the independent Student’s t-test or Mann-Whitney U test was employed, depending on the data distribution. Categorical variables were assessed using Pearson’s chi-square test. Spearman’s rank correlation analysis was conducted to examine the association between HIF-2α levels and the clinical or echocardiographic parameters. A two-sided p-value of less than 0.05 was deemed statistically significant for all tests.

## Results

### Baseline characteristics

This study included 69 patients with mitral stenosis (MS) and 69 healthy controls. The demographic and basic clinical characteristics of the two groups are shown in Table 1.

**Table 1:** Baseline characteristics and key laboratory findings for patients with mitral stenosis (MS) and healthy controls

Variable	Mitral Stenosis (*n* = 69)	Control (*n* = 69)	*p*-value
Age (years)	54 ± 15	50 ± 12	0.0503
Female sex – n (%)	50 (72.5%)	46 (66.7%)	0.459
HIF-2α, ng/ml	5.2 (4.0–7.1)	4.7 (3.8–5.8)	0.0275
hs-cTnT, ng/l	7.7 (4.1–14.0)	4.9 (3.0–7.3)	0.0328
NT-proBNP, pg/ml	679 (227–2517) †	–	–
Glucose, mg/dl	99 (90–120)	95 (87–109)	0.1996
HbA1c, %	5.7 (5.4–6.3)	5.7 (5.4–6.2)	0.7724
Urea, mg/dl	32 (25–38)	30 (24–35)	0.1526
Creatinine, mg/dl	0.87 (0.75–1.10)	0.84 (0.69–0.96)	0.0632
Albumin, g/l	40 ± 6.2	44 ± 3.0	0,0007
WBC count (×10³/µl)	8.0 (6.2–9.8)	7.3 (6.4–8.3)	0.3541
RBC count (×10⁶/µl)	4.6 (4.3–4.9)	4.8 (4.4–5.1)	0.0628
Platelet count (×10³/µl)	252 (190–300)	265 (226–325)	0.1121
Haemoglobin, g/dl	13 ± 1.9	14 ± 1.7	0,0086

Data are presented as mean ± SD or median (IQR), as appropriate. p-values refer to comparisons between the MS and control groups

*Abbreviations SD* standard deviation, *IQR* interquartile range, *hs-cTnT* high-sensitivity cardiac troponin T, *NT-proBNP* N-terminal pro-B-type natriuretic peptide, *WBC* white blood cell, * RBC* red blood cell

†NT-proBNP level was measured only in patients with MS (not in controls)

The mean age of patients with MS was 54 ± 15 years compared with 50 ± 12 years for controls (*p* = 0.0503), indicating a similar middle-aged profile in both groups. Females predominated in the MS (50 women, 72.5%) and control groups (46 women, 66.7%), with no significant difference in sex distribution (*p* = 0.459). Beta-blocker use was present in 60 patients and diuretic use was present in 56 patients in the MS group.

**Fig. 1:**
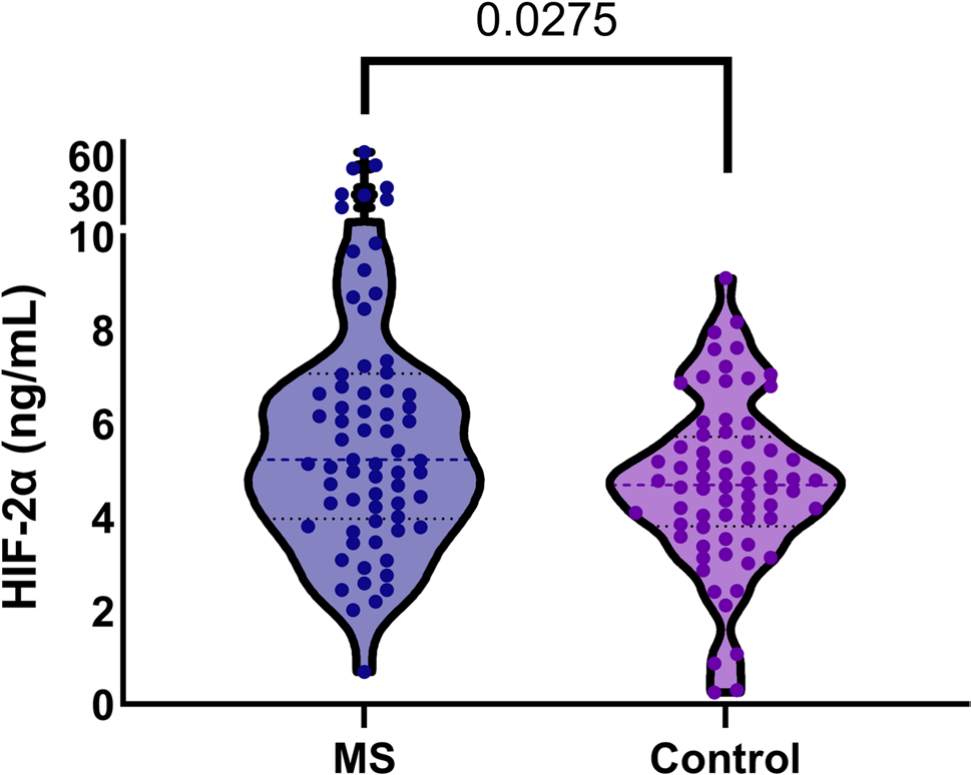
The HIF-2α levels of all participants are shown along with the median and interquartile range values for the mitral stenosis and control groups. Abbreviations: MS, mitral stenosis

Plasma HIF-2α concentrations were higher in patients with MS than in the controls. The median concentration in patients with MS was 5.2 ng/ml (IQR 4.0–7.1), with a mean ± SD of 9.2 ± 12.0 ng/ml, whereas controls had a median of 4.7 ng/ml (IQR 3.8–5.8) and a mean ± SD of 4.9 ± 2.6 ng/ml (*p* = 0.0275, Mann–Whitney U test). No significant correlation was observed between HIF-2α levels and age (Spearman’s rank correlation test, *p* = 0.391), and HIF-2α levels did not differ according to sex (Mann–Whitney U *test*,* p* = 0.305). Figure 1 illustrates the distribution of the HIF-2α levels in each patient group.

The median high-sensitivity troponin T (hs-cTnT) level in the MS group was 7.7 ng/l (IQR 4.1–14.0), compared to 4.9 ng/l (IQR 3.0–7.3) in the control group (*p* = 0.0328). NT-proBNP was only available for the MS group, with a median level of 679 pg/ml (IQR 227–2517). In the mitral stenosis group, haemoglobin (13 ± 1.9 g/dl vs. 14 ± 1.7 g/dl, *p* = 0.0086) and serum albumin levels (40 ± 6.2 g/l vs. 44 ± 3.0 g/l, *p* = 0.0007) were significantly lower than those in the control group. No statistically significant differences were observed in the other laboratory parameters.

Echocardiographic evaluation of the patients with mitral stenosis showed left atrial enlargement, elevated pulmonary arterial pressure, and reduced mitral valve area (Table 2).

**Table 2:** Key echocardiographic characteristics of patients

Parameter	Control Group (*n* = 69)	MS patients (*n* = 69)
Left atrial diameter (cm)	3.5 (2.8-4)	4.1 (3.6–4.8)
Mean transmitral gradient (mmHg)	-	7 (5–9.5)
Pulmonary arterial pressure (mmHg)	23 (18–26)	39 (34–47)
Mitral valve area (cm²)	5 (5–5)	1.6 (1.4–1.8)

Data are presented as the median (IQR)

Because no significant transmitral gradient was found in the control group, this value was not reported

*Abbreviations MS* mitral stenosis, * IQR* interquartile range

Based on ROC analysis, the optimal cut-off value for HIF-2α was 5.84 ng/ml, yielding a sensitivity of 46.38% and specificity of 76.81% (Youden index: 0.2319), reflecting limited discriminative capacity.

### Correlation of HIF-2α with disease severity and biomarkers

**Fig. 2:**
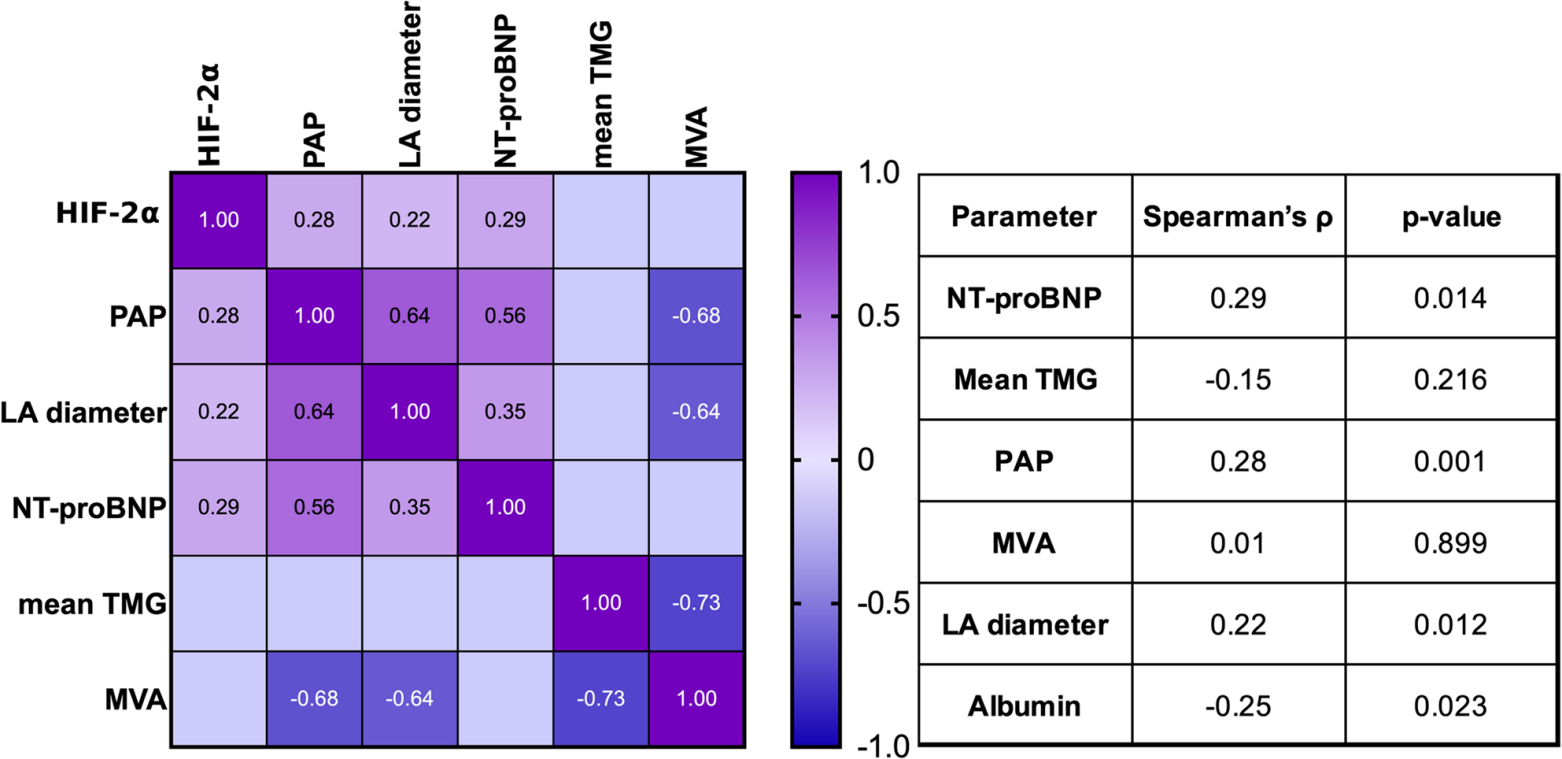
Spearman correlations between echocardiographic and biochemical parameters. The Spearman ρ values are shown in the heatmap, and cells with statistically non-significant correlations are left blank. The table on the right shows the correlation results for the relationship between the parameters and HIF-2α. Abbreviations: PAP, Pulmonary arterial pressure; NT-proBNP, N-terminal pro–B-type natriuretic peptide; LA diameter, left atrial diameter; mean TMG, mean transmitral gradient; MVA, Mitral valve area

In the mitral stenosis group (*n* = 69), we examined the association between HIF-2α level and various indicators of disease severity (Fig. 2).

**Fig. 3:**
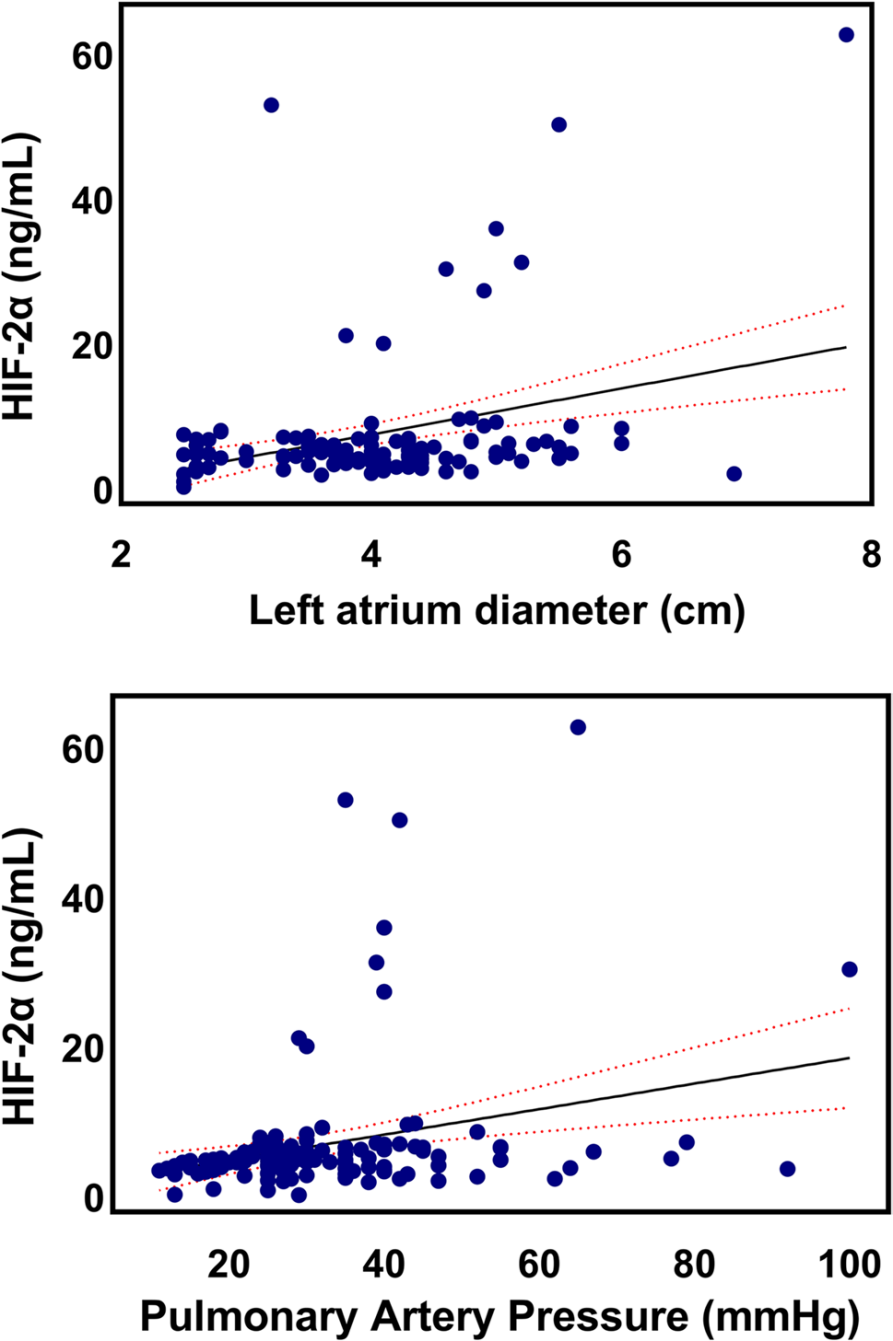
Scatter plots illustrating the relationship between circulating HIF-2α levels and pulmonary arterial pressure and left atrial diameter. Trend lines with 95% confidence intervals indicate the signalling of associations

HIF-2α levels demonstrated a weak but statistically significant positive correlation with systolic pulmonary artery pressure (Spearman’s ρ = 0.28, *p* = 0.001) and left atrial diameter (Spearman’s ρ = 0.22, *p* = 0.012). HIF-2α levels also positively correlated with NT-proBNP levels (Spearman’s ρ = 0.29, *p* = 0.014). An inverse correlation was observed between HIF-2α and serum albumin levels (ρ = − 0.252, *p* = 0.022). Figure 3 presents scatter plots of HIF-2α vs. PAP and the left atrial diameter with trend lines.

HIF-2α was not significantly correlated with the mitral valve area or mean transmitral gradient. When patients were stratified according to mitral stenosis severity as mild, moderate, or severe [7], no statistically significant differences were observed in HIF-2α levels between the groups.

## Discussion

This study demonstrates for the first time that circulating HIF-2α levels are higher in patients with mitral stenosis than in healthy individuals. HIF-2α showed a weak but significant positive correlation with systolic pulmonary artery pressure, NT-proBNP, and left atrial diameter and may be associated with the hemodynamic severity of the disease.

HIF-2α levels were not correlated with the anatomical severity of mitral stenosis, indicating that valve tightness was not directly related to HIF-2α levels. This suggests that HIF-2α elevation in MS is driven more by downstream hemodynamic consequences (such as pulmonary hypertension and possibly hypoxemia) than by valve narrowing.

It is important to consider the potential impact of medical treatments on our findings. Most patients with mitral stenosis were receiving diuretics and beta-blockers, which are expected to reduce atrial load and pulmonary congestion. However, HIF-2α levels remained significantly elevated compared to those in controls, suggesting that disease-related pathophysiology may outweigh the compensatory effects of standard medical therapy.

In the context of chronic MS, several factors contribute to HIF-2α stabilization and its release into circulation. First, long-standing pulmonary venous hypertension leads to pulmonary arteriolar remodelling and alveolar-capillary oxygen diffusion abnormalities, creating regions of relative hypoxia in the lungs. HIF-2α is predominantly expressed in vascular endothelial cells [8], and hypoxemia in pulmonary arteries upregulates HIF-2α expression in these cells [9]. HIF-2α is a critical driver of hypoxia-induced pulmonary hypertension. Studies in mice have shown that heterozygous HIF-2α deficiency protects against the development of pulmonary hypertension under chronic hypoxia, and endothelial cell–specific HIF-2α knockdown prevents hypoxia-induced vascular remodelling and increases pulmonary pressure [10]. Our findings align with these experimental observations, suggesting that HIF-2α reflects the degree of hypoxic pulmonary vascular response in MS patients.

Second, HIF-2α may be upregulated because of endothelial injury and inflammation triggered by rheumatic mitral valve disease. Shear stress can act as a mechanotransductive stimulus, leading to the activation of the HIF signalling pathway [11]. Although the primary pathology is valvular MS, severe MS can cause elevated pressure and turbulent flow, injuring the pulmonary endothelium. Injured or hypoxia-exposed endothelial cells stabilize HIF-2α, which then enters circulation. Therefore, HIF-2α levels in the blood may serve as a biomarker of endothelial dysfunction in the pulmonary vasculature and systemic circulation. It has been reported that HIF-2α activation under hypoxia can downregulate the endothelial tissue factor pathway inhibitor (TFPI), shifting towards a pro-thrombotic state [12]. There is a well-known risk of left atrial thrombus formation in patients with MS, particularly in those with AF. Although speculative, one might consider that elevated HIF-2α levels could contribute to a pro-coagulant milieu in MS (through reduced TFPI and potentially other coagulation pathways), thereby linking hypoxic signalling with thromboembolic risk.

The role of HIF-2α as a possible biomarker in MS can be compared with that of established markers such as natriuretic peptides. Plasma BNP levels are higher in patients with severe MS and are correlated with pulmonary artery pressure and symptoms [13]. In another study, MR-proANP and sCD146 levels decreased after successful percutaneous mitral commissurotomy, indicating that these biomarkers reflect hemodynamic improvements [14]. In our study, HIF-2α levels behaved in parallel with NT-proBNP levels; both were elevated in patients with MS compared to controls, and both were correlated with PAP. This suggests that HIF-2α, like BNP, responds to strain in the heart and the pulmonary vasculature. However, HIF-2α provides a different layer of information that is directly tied to hypoxia and angiogenic signalling, rather than myocardial wall stress. Thus, HIF-2α may integrate the effects of hypoxemic stress and vascular remodelling, which are not fully captured by natriuretic peptides.

Although hemoglobin and albumin levels were lower in the mitral stenosis group, the overall laboratory profile was broadly comparable between the groups, suggesting that the two groups had comparable systemic profiles and that differences in HIF-2α expression were not attributable to general metabolic or inflammatory disparities.

An ancillary finding was that high-sensitivity troponin T levels were modestly higher in patients with MS than in the controls. Chronic rheumatic MS has not been classically associated with significant troponin release; however, using contemporary high-sensitivity assays, minimal myocardial injury can be detected. Elevated troponin levels in patients with MS may result from chronic right ventricular pressure overload (causing subclinical RV ischemia), left atrial distension (leading to atrial myocardial stress), or concomitant coronary microvascular dysfunction due to low cardiac output. Previous studies on the role of troponin in valvular disease have shown mixed results [15]. Regardless, the difference in troponin levels in our data supports the notion that, even in chronic valvular disease, there can be ongoing minor myocardial injury. Troponin has proven prognostic value in aortic stenosis [16]; however, it remains to be seen whether a similar prognostic role exists for troponin in MS. Our study was not designed to evaluate outcomes, but the elevated hs-cTnT levels in patients with MS add to the evidence that MS is not a benign condition and causes measurable myocardial stress.

The study found no direct correlation between HIF-2α and mitral valve area, suggesting that HIF-2α serves as an indicator of the consequences rather than the anatomical obstruction of the valve itself. HIF-2α regulates tissue hypoxia and cellular response. HIF-2α levels may be more of an outcome/response indicator than those associated with anatomical pathologies. HIF-2α is also a molecule that can be affected by systemic hypoxia, inflammation, and genetic variations; therefore, the concentration of HIF-2α in circulation is related not only to heart valve anatomy, but also to the patient’s chronic hypoxic burden, pulmonary vascular response, accompanying lung or hematological conditions, and time-dependent adaptations. In light of these mechanisms, we interpret that HIF-2α does not reflect anatomical obstruction itself, but rather reflects the hemodynamic consequences caused by obstruction and tissue-level outcomes, such as pulmonary hypertension, hypoxia, and vascular remodelling.

The link between HIF-2α and pulmonary hypertension in patients with MS requires further investigation. HIF-2α and HIF-1α are the two main hypoxia-inducible factors isoforms with overlapping but distinct roles. The hypoxia response dynamics of HIF-1α and HIF-2α are different; HIF-1α is typically the first responder to acute hypoxia, driving glycolysis and short-term adaptive genes, whereas HIF-2α is involved in chronic hypoxia, angiogenesis, and erythropoiesis [17, 18]. In the context of chronic MS, hypoxia is not sudden systemic hypoxia but rather chronic pressure-induced hypoxia in the pulmonary vasculature and perhaps periodic systemic hypoxia during exercise. HIF-2α’s known functions align with the needs of a chronically hypoxic environment: it induces erythropoietin and VEGF [19], potentially aiding hypoxic tissue perfusion.

Preclinical studies have demonstrated that activation of hypoxia-inducible factors (HIF) can influence vascular tone and inflammatory responses by modulating extracellular nucleotide signaling [20, 21]. Additionally, HIF-regulated miRNAs are implicated in hypoxic response mechanisms, including angiogenesis, cell proliferation, apoptosis, and metabolic adaptation [17, 22]. Although these pathways were not evaluated in the present study, they warrant further investigation in future mechanistic studies.

The positive correlation between HIF-2α and PAP indicates that HIF-2α may be a mediator of adverse remodelling in pulmonary arteries due to MS. Elevated PAP in MS arises from a combination of passive backward pressure transmission, active pulmonary vasoconstriction, and vascular remodelling. Hypoxia is a potent stimulus for pulmonary vasoconstriction and arterial remodelling via the HIF pathway [10]. HIF-2α orchestrates the expression of genes that promote pulmonary arterial smooth muscle proliferation and vascular remodelling. For instance, Wang et al. demonstrated that HIF-2α drives the signaling of intercellular adhesion molecule-1 (ICAM-1) in endothelial cells, contributing to hypoxia-induced pulmonary hypertension [23]. In hypoxic settings, HIF-2α is instrumental in coordinating erythropoiesis, shaping inflammatory processes, and adapting cellular metabolism [6]. Taken together, these data suggest that HIF-2α elevation in patients with MS is not just an epiphenomenon, but may actively participate in the pathogenesis of secondary pulmonary hypertension. This raises an intriguing question: could therapies that modulate HIF-2α activity ameliorate pulmonary hypertension within MS patients?

Therapeutically, modulation of HIF signalling has emerged as a potential strategy for treating pulmonary vascular diseases [6, 24, 25]. Small-molecule HIF-2α inhibitors such as PT2385, PT2567, and PT2977 have shown efficacy in preclinical studies. For instance, in a rodent model of chronic hypoxic pulmonary hypertension, HIF-2α inhibition attenuated pulmonary vascular remodelling and right ventricular hypertrophy [26]. These observations implicate excessive HIF-2α activity in the pathogenesis of pulmonary hypertension and suggest that targeting this pathway could represent a future adjunctive approach for secondary pulmonary hypertension associated with MS. However, the potential role of such strategies warrants further evaluation in mechanistic and prospective clinical studies. Given that HIF signalling also mediates adaptive responses such as angiogenesis and metabolic adjustment, indiscriminate inhibition may have adverse effects. The broader concept of fine-tuning the hypoxic response remains attractive, with supportive evidence from conditions, such as high-altitude pulmonary edema and chronic mountain sickness, in which *HIF2Α* polymorphisms modify disease severity [27, 28].

Recently, it was shown that the transcription factor BMAL1 can form a transcriptionally active heterodimer with HIF-2α and reduce its ubiquitination of HIF-2α, thereby enhancing its stability [29]. This suggests that HIF-2α is involved in a complex transcriptional network. In this context, future studies examining HIF-2α together with BMAL1 and other components of the HIF signaling pathway, such as HIF-1α, as well as downstream angiogenic effectors, including VEGF, with a focus on their regulatory interactions within broader molecular networks, may provide valuable insights into pulmonary hypertension.

### Limitations

This study was limited by its single-center design, moderate sample size, cross-sectional assessment of circulating HIF-2α levels without tissue correlation, and inclusion of only patients with rheumatic mitral stenosis. Precise information on the time since diagnosis was not available for all patients; therefore, echocardiographic parameters were used to reflect the baseline disease severity. The lack of detailed subgroup analysis based on specific dosages and treatment durations should be considered when interpreting our results. HIF-2α was assessed at a single time point, and pre- and post-treatment measurements were not available. Therefore, the potential role of HIF-2α as a marker of therapeutic response could not be evaluated. Considering these limitations, HIF-2α should be studied in larger cohorts to determine whether it adds predictive value for outcomes, such as symptom progression, development of severe pulmonary hypertension, right heart failure, or the need for intervention. Moreover, HIF-2α levels may decrease after effective treatment of MS (e.g., percutaneous balloon commissurotomy or valve replacement) if the hypoxic stimulus is relieved, which can be evaluated in future longitudinal studies.

## Conclusion

To our knowledge, this is one of the first focused investigations of HIF-2α in mitral valve disease, providing preliminary insights into the biochemical profile of MS. HIF-2α levels were higher in patients than in controls but were related only to NT-proBNP, pulmonary artery pressure, and left atrial diameter, not to the anatomical measures of stenosis severity. These findings indicate that HIF-2α may reflect certain aspects of the pathophysiological burden of MS. However, these findings represent observational associations and do not imply a causal relationship. HIF-2α may reflect aspects of hypoxia-related and hemodynamic burden in MS, but its functional role in pulmonary hypertension cannot be concluded from the present data, and its role as a biomarker of disease severity remains unclear. Further studies are required to clarify the clinical relevance of HIF-2α in valvular heart disease and pulmonary hypertension.

## Data Availability

To access the data that forms the basis of this study’s results, interested parties should contact the corresponding author upon reasonable request.

## Abbreviations

ELISA: Enzyme-linked immunosorbent assay
EPAS1: Endothelial PAS domain protein 1
HIF: Hypoxia-inducible factor
HIF-2α: Hypoxia-inducible factor-2 alpha
hs-cTnT: High-sensitivity cardiac troponin T
IQR: Interquartile range
MS: Mitral stenosis
MVA: Mitral valve area
NT-proBNP: N-terminal pro–B-type natriuretic peptide
PAP: Pulmonary artery pressure
SD: Standard deviation
TFPI: Tissue factor pathway inhibitor
